# Positional Differences in Jump Loads and Force and Velocity Metrics Throughout a 16-Week Division I Volleyball Season

**DOI:** 10.1155/tsm2/5933923

**Published:** 2024-12-18

**Authors:** Gabriel J. Sanders, Stacie Skodinski, Damjana V. Cabarkapa, Mason Howard, Dimitrije Cabarkapa, Corey A. Peacock

**Affiliations:** ^1^Department of Exercise Science, University of Cincinnati, Cincinnati, Ohio, USA; ^2^Department of Strength and Conditioning, University of Cincinnati, Cincinnati, Ohio, USA; ^3^Department of Health, Sport & Exercise Sciences, University of Kansas, Lawrence, Kansas, USA; ^4^Department of Kinesiology and Health, University of Central Florida, Orlando, Florida, USA; ^5^Department of Kinesiology, Nova Southeastern University, Fort Lauderdale, Florida, USA

## Abstract

The study quantified total and high-intensity jump counts and assessed neuromuscular performance through countermovement jump (CMJ) force and velocity metrics by position. Twelve Division I female athletes (19.6 ± 1.3 years; 182.7 ± 6.5 cm) were included in the 16-week study using wearable microsensors to monitor daily jump loads. CMJ tests were conducted twice weekly using dual force plates to measure force and velocity metrics. There were significant main effects of position (*p* ≤ 0.001) for jump and force plate metrics. Middle blockers accumulated significantly more jump counts of 38.1 cm or higher (jumps 38+; 65.4 ± 39.2 counts) and jump counts of 50.8 cm or higher (jumps 50+; 39.5 ± 32.7 counts) compared to outside hitters (jumps 38+; 39.4 ± 25.9 counts and jumps 50+; 15.0 ± 15.6 counts) and opposite hitters (jumps 38+; 47.9 ± 24.1 counts and jumps 50+; 29.7 ± 18.1 counts), while setters had the fewest high-intensity jump counts (jumps 38+; 19.0 ± 16.6 counts and jumps 50+; 0.4 ± 0.8 counts). Middle blockers had the highest CMJ height (36.1 ± 6.4 cm), deepest CMJ depth (−41.7 ± 6.4 cm) and peak (2.75 ± 0.22 m/s) and average (1.49 ± 0.08 m/s) propulsion velocities (2.75 ± 0.22 m/s). Meanwhile, setters had significantly greater braking RFD (7839 ± 2617 N), average (1698 ± 223 N) and peak braking force (2061 ± 248 N), and average (1446 ± 88 N) and peak propulsion force (1994 ± 213 N), compared to all other positions. Opposite and outside hitters' data fell between setters and middle blockers. Regardless of position, neuromuscular performance fluctuates during the season and there are noticeable positional differences in jump loads and force and velocity metrics.

## 1. Introduction

Volleyball requires athletes to execute frequent and repetitive high-intensity jumps with minimal rest intervals [1–3]. Monitoring jump loads and jump load intensity is essential to understand the varying stress levels placed on athletes, particularly with respect to positional differences. The quantity and intensity of jumps vary due to factors such as practice periodization day, competition, and volleyball position [4–7]. Previous research shows that middle blockers (MBs) and outside hitters (OHs) consistently perform more high-intensity jumps than setters (Ss) and opposite hitters (OPPs), with MB and OPP accumulating the highest volumes of intense jumps, while Ss typically have the least [2, 5–9]. The physical stress from an excessive number of high-intensity jumps has demonstrated a significant impact on inducing fatigue and increasing the risk of injury [8, 10–12]. As a result, it is feasible to suggest that MB and OPP are more susceptible to neuromuscular fatigue and potential overuse injuries due to their consistently higher volumes of intense jump loads, underscoring the importance of position-specific monitoring and recovery strategies.

Previous studies have utilized countermovement jump (CMJ) tests, performed on force plates to assess neuromuscular fatigue, as CMJ has been proven to be a valid and reliable method to evaluate alterations in lower-body power and neuromuscular fatigue [10–14]. The use of force plates allows the analysis of both concentric (i.e., propulsion) and eccentric (i.e., braking) function and their relationship with neuromuscular performance [11, 12]. Utilizing validated force plates for objective measures of neuromuscular performance may be an ideal method of monitoring fatigue (i.e., acute or chronic fatigue), especially when objectively measured jump loads are assessed simultaneously. Assessing an internal and external load throughout an entire volleyball season can help practitioners better understand the extent to which high-intensity jump loads affect neuromuscular performance.

Prior to assessing fatigue, it is imperative to define high-intensity jump loads as there are many different wearable microsensor technologies available and these manufacturers categorize jump loads and intensities differently, which matter when assessing and reporting fatigue [4, 15–17]. Different devices quantify jump loads differently due to their algorithms impacting the measurement of jump heights [17, 18]. For example, two widely adopted devices in volleyball quantify high-intensity jump loads differently with one manufacturer defining high-intensity jumps as > 40 cm (equates to 15.7 inches), while a different manufacturer categorizes high-intensity jumps as > 20 inches (equates to 50.8 cm) [8, 18]. Identifying which jump intensity threshold best correlates with neuromuscular performance or recovery indices is not well-established. The categorization threshold difference should be considered specifically expressed when assessing positional differences in jump loads and intensities. As previously mentioned, positional differences exist in high-intensity demands among volleyball athletes, with MBs, OHs, and OPPs traditionally accumulating a greater number of high-intensity jumps than Ss [5–8]. However, it is not well-established if various positions experience neuromuscular fatigue differently based on jump loads throughout a season and what degree of high-intensity jumps impacts jumping force and velocity.

Understanding jump load requirements and their impact on neuromuscular fatigue is crucial for optimizing volleyball performance, as jump monitoring has been shown to improve training periodization, enhance performance, and help prevent injuries [4, 8, 19–21]. However, research in elite women's volleyball, relative to men, is sparse regarding how jump loads at various intensities alter neuromuscular performance throughout a season and if there are identifiable differences between volleyball positions. Therefore, the purpose of this study is to quantify total jump counts, as well as jump counts exceeding 38 cm (jumps 38+) and 50 cm (jumps 50+) and to assess neuromuscular performance by analyzing force and velocity metrics from CMJ tests in relation to jump counts over the course of the season. The hypothesis is that positional differences will exist in jump loads, force, and velocity, however, regardless of position, athletes accumulating greater high-intensity jump counts will experience increased neuromuscular fatigue, as indicated by reduced force production on the CMJ test, compared to positions with fewer high-intensity jump counts.

## 2. Materials and Methods

The retrospective study analyzes routinely collected data from a National Collegiate Athletic Association (NCAA) Division I volleyball team, including jump load and force plate metrics gathered over the course of a 16-week season. Division I women's volleyball athletes were studied based on their positions using wearable microsensor technology to monitor their daily jump loads throughout an entire competitive season (2 weeks of preseason camp and 14 weeks of the season). Data were collected on OHs (*N* = 4), OPPs (*N* = 2), MBs (*N* = 4), and Ss (*N* = 2). The devices tracked the frequency and intensity of jump loads during practices and matches. Neuromuscular fatigue was also assessed via twice weekly CMJ tests on a computerized dual force plate allowing concentric (propulsion) and eccentric (braking) forces and velocity to be measured.

### 2.1. Participants

Twelve NCAA Division I female volleyball athletes (age: 19.6 ± 1.3 years; height: 182.7 ± 6.5 cm) satisfied the inclusion criteria for this retrospective analysis. The inclusion criteria required female volleyball athletes aged 18–23 to be medically cleared, to have worn a monitoring device consistently throughout the season, and to have actively participated in either games or practices within three days prior to each CMJ test; athletes not meeting these conditions were excluded. During each practice and match competition, athletes wore a waist-mounted microsensor device to monitor movement, specifically jumps. Then, athletes performed CMJs twice per week on a dual force plate, which measured concentric (propulsion) and eccentric (braking) forces [22]. Weight (mass) was not reported due to university constraints; however, it was measured before each CMJ by the force plate device for metrics requiring weight-derived values. These measurements were part of the university's athlete monitoring protocol and normal athletic activities. All athlete data were deidentified prior to research access and the university's Institutional Review Board (IRB) approved the retrospective analysis. Prior to participation in varsity sports, athletes received medical clearance from the team physician and signed an informed consent form.

### 2.2. Protocol

The study retrospectively evaluated data from a team of NCAA Division I female volleyball athletes throughout an entire season, using the inertial measurement unit, a microsensor device designed with a 3-axis accelerometer, gyroscope, and magnetometer (VERT 3, Fort Lauderdale, Florida, USA) [2, 6, 15, 17]. The device was worn at the top of the iliac crest and secured with a tight band according to the manufacturer's guidelines. The wearable device was worn during each practice and game by each athlete for the season. In addition to daily monitoring, neuromuscular performance was assessed for each athlete by performing a CMJ test, twice weekly. These tests were conducted using a validated, portable dual force plate system (Hawkin Dynamics, Westbrook, Maine, USA), which operates at a sampling rate of 1000 Hz [22, 23]. The CMJ assessments were performed at the beginning of the week before the first practice on Mondays, and the second CMJ tests were completed again the day before a home game or before traveling for an away competition, which occurred on a Thursday or Friday. The CMJ tests on the force plate were always completed prior to any type of warmup to prevent neural stimulation that could enhance CMJ performance and obscure indicators of neuromuscular fatigue [24]. During training camp, CMJ tests were conducted daily. Before each jump, athletes were required to stand motionless on the force plates with their hands on their hips. The force plate system measured the athlete's weight in Newtons and, upon completion, the software would emit a beep to signal readiness for the jump measurement. Following the coach's instruction, the athlete would then perform a maximal effort CMJ with hands remaining on their hips. This procedure was repeated once for a total of two jumps per session. These CMJ tests were conducted twice weekly, with weight measurements taken by the force plate to facilitate power calculations.

### 2.3. Data Analysis

**Figure 1:**
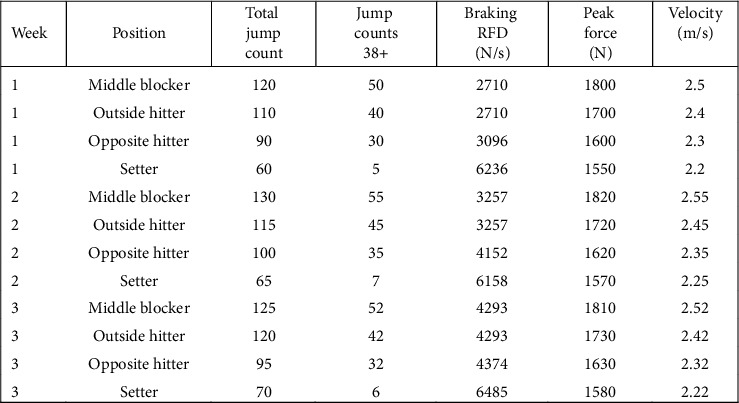
The example data file demonstrates how jump load data and force plate metrics from CMJ tests were merged and aligned weekly to assess average values for each volleyball position throughout the season. By synchronizing jump load data (including total and high-intensity jump counts) with neuromuscular performance metrics (e.g., force and velocity outputs), the analysis captures the week-to-week impact of training load on neuromuscular fatigue. This alignment allows for a clearer comparison of workload variations and their effects on performance across positions, enhancing the statistical power of the study.

After every practice and match, data from the wearable devices were downloaded using the software provided by the manufacturer. The force plate data from the CMJ tests were measured with the validated Hawkin Dynamics software [22, 23]. The jump loads were monitored daily totaling 1544 recorded observations including practices (*N* = 60), scrimmages (*N* = 2), and competitive matches (*N* = 28) from the athletes. There were 2 weeks of preseason training that led up to the first week of training for a match week. The total session count (1544) included all types of training activities, such as team practices, individual sessions, walkthroughs, and double training sessions during preseason. In compliance with the university's athletic agreement, only the total number of recorded sessions is provided without specifying injury data. The jump loads and CMJ test results were averaged to establish weekly averages. First, the data were organized by weekly averaged jump loads and weekly force plate metrics over a 16-week period and compared across four different volleyball positions (MBs, OHs, OPPs, and Ss). Then, the jump load data were merged with the force plate data so that the weekly jump data corresponded to the same weekly force plate data. The weekly timeline for all data was from Monday to Sunday. Averaging jump loads and CMJ tests on a weekly basis reduces the influence of day-to-day variability in jump loads and other extraneous variables such as poor nutrition, sleep, and recovery. The weekly aggregation allows for a more consistent and reliable assessment of long-term trends in jump loads and neuromuscular performance. In addition, by aligning the temporal scale of jump loads and force plate data from the CMJ tests, the analysis gains statistical power, providing a clearer understanding of how changes in training load impact neuromuscular performance weekly for each position (Figure 1).

The CMJ test metrics included the following: jump height (cm) was calculated as the difference between the peak vertical displacement during takeoff and the standing height prior to the jump and CMJ depth (cm) was measured as the vertical distance the athlete lowered during the eccentric phase of the jump. The braking rate of force development (RFD) was determined as the rate of change in force during the eccentric phase of the jump and expressed in Newtons per second (Newton per second). Average braking force (Newtons) and peak braking force (Newtons) were recorded, representing the mean and maximum forces applied during the deceleration phase of the jump. For the propulsion phase, average propulsion force (Newtons) and peak propulsion force (Newtons) were calculated, representing the mean and maximum forces generated during the concentric phase leading to takeoff.

Velocity metrics were also captured and measured as meters per second (m/s), with average braking velocity (m/s) and peak braking velocity (m/s) representing the mean and maximum downward velocities during the eccentric phase. Similarly, average propulsion velocity (m/s) and peak propulsion velocity (m/s) were recorded to assess the mean and maximum velocities during the concentric phase of the jump. These metrics were used to evaluate the athletes' neuromuscular performance from four different positions throughout the 16-week season.

### 2.4. Statistical Analysis

Descriptive statistics (means ± standard deviations) were used to analyze all jump load and force plate metrics for each position across a 16-week season. Mixed-effects models were utilized to assess the main and interaction effects of two fixed factors: volleyball position (MBs, OHs, OPPs, and Ss) and time (16 weeks), on all dependent variables related to jump loads and force plate metrics from the CMJ tests. The dependent variables included total jump counts, high-intensity jump counts (jumps 38+ for jumps > 38.1 cm and jumps 50+ for jumps > 50.8 cm, categorized by the manufacturer), CMJ height, CMJ depth, as well as all force and velocity metrics. The use of mixed-effects models was necessary due to the hierarchical structure of the data, with repeated measures across weeks nested within each player, and the variability expected between different position groups [25]. This approach accounts for the interdependence of observations within players and positions over time, allowing for both fixed and random effects to be properly modeled.

To interpret the magnitude of significant effects, Cohen's partial eta squared (*ηp*^2^) was calculated as an effect size measure for each fixed factor and interaction. Cohen's benchmarks (small: *ηp*^2^ ≈ 0.01, medium: *ηp*^2^ ≈ 0.06, and large: *ηp*^2^ ≈ 0.14) were used to evaluate the strength of these effects, providing additional context to the statistical significance [26]. Post hoc pairwise comparisons were conducted using the Bonferroni method to adjust for multiple comparisons.

Lastly, correlations were used to explore relationships between high-intensity jump counts (jumps 50+) and neuromuscular performance indicators when significant position-by-week interactions were presented. By assessing overall team and positional correlations for variables with significant position-by-week interactions, it was feasible to assess if changes in high-intensity jump counts were associated with changes in neuromuscular performance indicators. All statistical analyses were performed using IBM SPSS Statistics software Version 29.0 (IBM Corp., Armonk, New York), with the threshold for statistical significance set at *p* ≤ 0.05.

## 3. Results

### 3.1. The Effect of Four Position Groups

Mixed-effects results are reported in Table 1 revealing there was a significant main effect of position on all jump and force plate-based metrics. In Tables 2 and 3, positional data are reported for all jump and force plate metrics.

#### 3.1.1. Jump Metrics

MBs had 42% more total jump counts compared to OHs and 26% more than OPPs but 33% less than Ss. MBs also performed 66% more jumps of 38+ cm in height and 164% more jumps of 50+ cm in height than Ss. OPPs recorded similar high-intensity jump percentages to MBs, while Ss had less than 1% of their total jumps in the 50+ cm category. Furthermore, MBs demonstrated the highest CMJ height, averaging 36.1 cm, which was 20% higher than OHs, 29% higher than OPPs, and 32% higher than Ss. CMJ depth also varied by position, with MBs showing the deepest CMJ depth (−41.7 cm), which was 5% greater than OHs and 21% deeper than both OPPs and Ss, which had nearly identical values. These findings emphasize the positional differences in jump performance, with MBs and OPPs experiencing significantly greater demands compared to Ss.

#### 3.1.2. Force Metrics

A large, significant main effect of position was observed for all force-based metrics. Ss had significantly greater force metrics than all positions and notably, Ss also had minimal high-intensity jump counts on a daily and weekly basis. Then, MBs exhibited higher average and peak braking and propulsion forces compared to OHs and OPPs. The braking RFD was 41% greater in Ss and Ss recorded a significantly greater braking RFD than all other positions.

#### 3.1.3. Velocity Metrics

There were small-to-medium significant main effects of position on all velocity metrics. All positions had significantly greater average and peak braking velocities than OHs. MBs had 13% higher average propulsion velocity than OPPs and 3.4% higher than OHs and Ss. The difference in braking velocity between positions was smaller, with MBs showing only a 7.1% increase in average braking velocity compared to OPPs and 2% compared to OHs. These results demonstrate that MBs have the highest overall velocity outputs.

### 3.2. The Effect of a 16-Week Season

Mixed-effects results are reported in Table 1, showing small-to-medium significant main effects of the week on all jump-based and most force-based and velocity-based metrics, except CMJ depth and average propulsion force. In Supporting Table 1, week-to-week data for all jump, force, and velocity metrics are provided as mean ± SD.

#### 3.2.1. Jump Metrics

There were noticeable changes in total jump counts throughout the 16-week season, with up to a 47% increase in the weeks with the highest jump loads (Weeks 11, 13, and 15) compared to the lowest Week 12 totals. Jump counts of 38+ cm also varied significantly, with moderate differences between the highest 62.8 average counts at Week 15–30 average counts during Week 12. Jump counts of 50+ showed even larger fluctuations, with some weeks (Week 3) athletes averaging up to 53.8% greater high-intensity jump counts than in Week 6. CMJ height remained relatively stable across the weeks with only a small difference between Weeks 3 and 8. There were no significant changes in CMJ depth over the season.

#### 3.2.2. Force Metrics

The main effect of the week on force metrics revealed specific changes over the season. Braking RFD was significantly lower in Week 1 compared to Weeks 3, 5–10, 12, and 14–16. The average braking force in Week 1 was significantly lower than in all weeks except Weeks 2 and 4. The peak braking force was lower in Week 1 compared to Weeks 3 and 5–16. Peak propulsion force was significantly lower in Week 1 than in Weeks 5, 9, 12, 15, and 16, while average propulsion force showed no significant differences between weeks.

#### 3.2.3. Velocity Metrics

The main effect of the week on velocity metrics showed distinct changes. The average braking velocity was significantly lower in Week 1 compared to Weeks 3, 5, 7–10, 12, 13, 15, and 16. Peak braking velocity in Week 1 was lower than in Weeks 5, 7–10, 12, 13, 15, and 16, and Weeks 3 and 4 differed from Week 12. The average propulsion velocity was lower in Week 1 compared to Weeks 3, 5, 7, 9, 12, and 16, and Week 2 was different from Week 12. Peak propulsion velocity only showed differences between Weeks 3 and 8.

### 3.3. Four Position Groups by 16-Week Interactions

**Figure 2:**
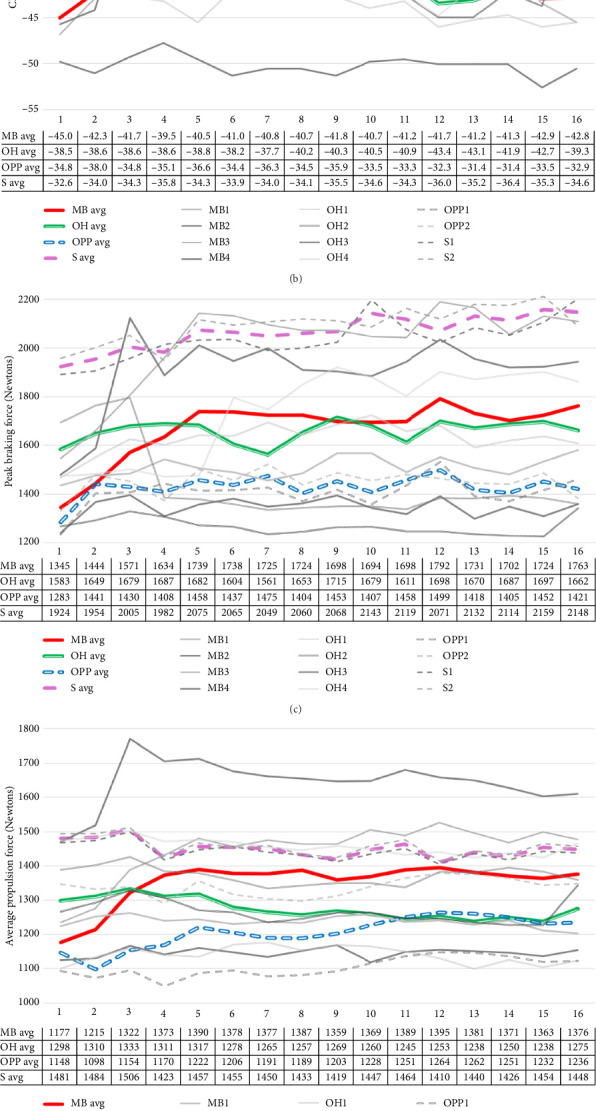
The graph illustrates jump counts for jumps 50.8 cm or higher, CMJ depth, peak braking force, average propulsion force, and peak braking velocity by position and individual throughout the 16 weeks of the season. Significant interactions for the graphs are reported in Table 1. (a) Weekly average 50+ jump counts by position (color lines) and individual athletes (gray lines). Data show the mean 50+ jump counts. (b) Weekly CMJ depth measures by position (color lines) and individual athletes (gray lines). Data show the mean CMJ depth. (c) Weekly peak braking force by position (color lines) and individual athletes (gray lines). Data show the mean force in Newtons. (d) Weekly average propulsion force by position (color lines) and individual athletes (gray lines). Data show the mean force in Newtons. (e) Weekly average peak braking velocity by position (color lines) and individual athletes (gray lines). Data show the mean velocity in meters per second.

There were five significant position-by-week interactions with medium effects (see Table 1). High-intensity jumps (50+ cm) exhibited a significant interaction, indicating that the positional differences in jump counts varied across the weeks.  Figure 2(a) shows 50+ cm jump counts over 16 weeks for volleyball positions, with MBs having the highest counts, peaking at 66.9 counts on average in Week 1, while OPPs had steady but lower counts. OHs and Ss had significantly fewer high-intensity jumps, with Ss consistently near zero. In addition, a significant interaction was found for CMJ height, and Figure 2(b) shows that MBs consistently demonstrated the deepest CMJ depths, averaging around −45 cm, while OPPs had shallower depths around −34 cm, and Ss recorded the shallowest depths near −33 cm throughout the season. Among force-based metrics, peak braking force and average propulsion force showed significant position-by-week interactions. Figures 2(c) and 2(d) reveal that Ss consistently produced the highest peak braking force and average propulsion force throughout the season, with both metrics reaching their peak around Weeks 8–9. MBs displayed moderate peak braking force but had an increasing trend in average propulsion force, peaking around Week 5. OPPs recorded the lowest values for both peak braking force and average propulsion force, remaining relatively stable with minimal fluctuations across the season. OHs maintained midrange values for both metrics, showing more variation in peak braking force than in propulsion force. Peak braking velocity was the only velocity metric demonstrating a significant interaction.  Figure 2(e) shows that OPPs had the most noticeable changes in peak braking velocity throughout the season, with significant fluctuations. In contrast, MBs and OHs remained relatively stable, while Ss showed a steady decline in braking velocity as the season progressed.

### 3.4. Correlations

As a team throughout the season, there were significant negative correlations between jump counts of 50+ and countermovement depth (*r* = −0.411, *p* < 0.001), peak braking force (*r* = −0.511, *p* < 0.001), average propulsive force (*r* = −0.404, *p* < 0.001), and peak braking velocity (*r* = −0.149, *p* < 0.001). The negative correlations indicate that as high-intensity jumps increased, CMJ depth, peak braking force, average propulsion force, and peak braking velocity decreased.

The positional correlations between jump counts of 50+ and CMJ variables revealed distinct patterns for each position (Table 4). For MB, jump counts of 50+ showed moderate to strong negative correlations with countermovement depth, peak braking force, and average propulsive force, with a weaker but significant correlation with peak braking velocity. OHs displayed weaker yet significant negative correlations with countermovement depth, average propulsive force, and peak braking velocity but no significant correlation with peak braking force. OPPs showed generally weak, nonsignificant correlations with all CMJ variables. Ss have weak but significant negative correlations with countermovement depth and peak braking velocity, with no significant correlations for peak braking force or average propulsive force. Overall, MBs exhibit the strongest associations, while OPPs show the weakest.

## 4. Discussion

This study is the first to examine how total jump loads and high-intensity jump loads vary between four different volleyball positions and how these loads impact CMJ test performance throughout a 16-week season. As anticipated, each position accumulated unique jump loads and intensities that vary weekly. Ss recorded significantly more total jumps but fewer high-intensity jumps at 38.1 cm and 50.8 cm or higher. MBs and OPPs performed a similar proportion of their total jumps at the highest intensity level (categorized as jump counts of 50+). Specifically, 44.6% of all jumps by MBs and 44.7% of all jumps by OPPs were at this high intensity. In contrast, Ss only had 0.3% of their total jumps at this same high-intensity level, indicating that Ss rarely perform jumps of this intensity compared to MBs and OPPs. Notably, the Ss produced a significantly greater amount of braking and propulsive force than all other positions, likely due to minimal weekly high-intensity jumps.

While jump counts differed, the findings related to the position-specific differences in total and high-intensity jump loads obtained in the present investigation were similar to the ones observed in the previous scientific literature [6–8]. Specifically, when examining a cohort of male professional volleyball players across the entire competitive season, Skazalski, Whiteley, and Bahr [6] showed that Ss had the highest volume (∼120 jumps per session) and frequency (∼90 jumps per hour) of the jumps during both training sessions and games, when compared to the other positions on the team (e.g., OHs, MBs, and OPPs). However, the majority of the jumps that Ss performed were at lower heights (∼40% of their maximum jump height). Similar observations were made by Vlantes and Readdy [7], who revealed that Ss had the highest jump loads (∼222 jumps), followed by MBs (∼135 jumps) and OHs (∼67 jumps). These findings directly align with the current results showing that Ss on the NCAA Division I female volleyball team had the highest total jump load but the least high-intensity jumps (i.e., jump counts of 38+ and jump counts of 50+). This can be attributed to the unique tactical and technical demands placed on the Ss and their responsibilities on the court. For example, Ss are required to manage the teams' offensive strategies and cover more ground to get into the optimal setting position [27], while the MBs engage in more high-intensity jumps due to their roles in blocking and attacking [8]. Therefore, it is critical for practitioners to take into consideration the position-specific, or more precisely jump load–specific, differences when tailoring training regimens to meet the unique demands of each athlete.

The corollary findings suggest that frequent high-intensity jumps (jumps 50+) are negatively correlated with CMJ metrics, including countermovement depth, braking force, and propulsive force, indicating potential neuromuscular fatigue across volleyball positions. This relationship suggests that high-intensity jump loads may compromise performance by reducing an athlete's ability to generate force and maintain effective jump mechanics. Cormie, Mcguigan, and Newton [28] found that training adaptations in the eccentric phase particularly increased musculotendinous stiffness and improved force transmission, contributing to enhanced concentric force output and overall jump performance. However, when athletes repeatedly perform high-intensity jumps without adequate recovery, these adaptations can be strained, potentially reducing braking and propulsive forces, as our study suggests. This diminished force production aligns with indicators of neuromuscular fatigue, where the stretch-shortening cycle benefits are compromised, resulting in less effective force transmission and power output [28, 29]. This pattern aligns with typical fatigue responses where athletes experience diminished ability to absorb and produce force, particularly in actions relying on the stretch-shortening cycle. Thus, the negative relationship observed reinforces that high-intensity jumps affect neuromuscular performance, necessitating jump load–specific recovery strategies to mitigate fatigue throughout the season.

The majority of the previous research investigations on position-specific differences during the CMJ have primarily reported the outcome metrics, such as vertical jump height [5, 30]. However, to the best of our knowledge, this is one of the first studies to comprehensively analyze position-specific differences in jump loads and neuromuscular performance of force and velocity during both braking and propulsive phases of the jumping motion within a cohort of collegiate female volleyball players during a competitive season. It was observed that Ss had significantly greater braking RFD, propulsive force, and brake force when compared to all the other positions (i.e., MBs, OHs, and OPPs), as well as lower CMJ depth and vertical jump height than the MBs and OHs. These discrepancies between positions may be largely attributed to the previously discussed game demands that require Ss to perform quick, precise, and controlled movements rather than high-intensity jumps (e.g., blocking and attacking), as well as the differences in the anthropometric characteristics (e.g., body height and body mass) that have been previously reported in the scientific literature [31]. However, future research on this topic is warranted to obtain a better understanding of the underlying biomechanical and physiological factors that may contribute to these position-specific alterations in CMJ performance, especially within the female athlete population.

There was a general trend of progress in neuromuscular performance from Week 1 to Week 16, but the improvements did not occur in a consistent, week-to-week linear fashion. Instead, performance metrics showed ups and downs, reflecting periods of both gains and plateaus, with an overall positive trend across the 16-week season. This indicates that while neuromuscular performance improved, the progress was irregular rather than steadily increasing each week. Specifically, athletes exhibited a significant increase in braking RFD, average and peak brake, and propulsive force and velocity by Week 16. Similar observations were made by Cabarkapa et al. [11], where female volleyball athletes playing at the NAIA level of competition significantly increased their mean and peak eccentric power and velocity. However, no significant changes were noted during the concentric (i.e., propulsive) phase of the jumping motion [10]. In addition, the aforementioned findings seem to be contradictory to the ones obtained by Philipp et al. [32], where no significant alterations in the countermovement vertical jump performance have been observed pre–postcompetitive season in male collegiate basketball players. However, the authors detected notable neuromuscular performance improvements during a transition period from preseason to nonconference, with all metrics returning back to baseline (i.e., preseason values) by the end of the season [32]. While this topic warrants further investigation, the aforementioned discrepancies can be primarily attributed to the differences in sports (basketball vs. volleyball), competitive levels (NAIA vs. NCAA), as well as sex-specific differences (male vs. female).

The results also highlight positional differences in jump loads, CMJ depth, and force and velocity metrics across the season. MBs consistently had the highest number of 50+ cm jumps, peaking at 67 counts in Week 1 (which is preseason training camp), while OPPs followed with relatively high counts, and Ss had the lowest jump loads. These differences are reflected in CMJ depth, with MBs demonstrating the deepest jumps (−45 cm) compared to Ss, who had the shallowest (−33 cm). In terms of force metrics, MBs also showed higher average propulsion force, peaking at 1390 N, though Ss consistently exhibited the highest overall propulsion force despite their lower jump loads. These findings suggest that higher jump loads in MBs and OPPs lead to greater neuromuscular demands, while Ss maintain high force outputs with fewer high-intensity jumps, thus the least neuromuscular demands throughout the season.

While this study provides valuable insights into the variability of jump loads and CMJ test performance across four volleyball positions, it does have limitations. First, the data were collected from a single team, with a sample of 12 athletes. It would be beneficial to assess these changes across multiple Division I volleyball teams to enhance generalizability. In addition, CMJ tests were conducted at least twice a week but more precise neuromuscular fatigue measures may require daily force plate assessments to fully capture the impact of daily jump loads, particularly between games and practices. However, the logistical challenges of collecting data from different teams and conducting daily CMJ tests, given the varying schedules, practice times, and travel demands, make such an approach difficult to consistently implement. In addition, the CMJ tests were administered before practices, but quick assessments after practices and games could offer practitioners valuable neuromuscular performance information for optimizing recovery throughout the season. Although postgame or postpractice assessments pose challenges, they could be crucial for refining recovery strategies. Future research should also consider investigating other contributing factors to neuromuscular fatigue, such as distance traveled, nights away from campus, and even heart rate variability. Understanding athlete neuromuscular fatigue by utilizing objective measures can enhance coaching strategies and improve periodization protocols for optimal training.

## 5. Conclusions

The study highlights position-specific differences in jump loads, which lead to variations in neuromuscular performance. For instance, CMJ depth variability reached 18.7% across positions, with the strongest negative correlations between high-intensity jumps (jumps 50+) and four CMJ variables observed when analyzed by position. In contrast, team-level analysis yielded weaker correlations and no significant differences. This suggests that CMJ test metrics should ideally be tailored to each position or even be jump load–specific, meaning that an individual athlete's CMJ performance, neuromuscular function, and fatigue are assessed based solely on their unique jump loads. This approach is feasible if daily data on both jump loads and CMJ performance are collected throughout a season. Such position-specific and jump load–specific assessments offer valuable insights that can inform targeted strategies to optimize neuromuscular performance and reduce fatigue.

## Figures and Tables

**Table 1:** Mixed-effects results and effect sizes for all dependent variables.

	Main effects of position				Main effects of a week				Position ∗ week interaction			
	*F*	*p* value	*ηp* ^2^	Effect	*F*	*p* value	*ηp* ^2^	Effect	*F*	*p* value	*ηp* ^2^	Effect
Jump-based metrics												
Total jump counts	107.71	< 0.001	0.179	Large	10.08	< 0.001	0.093	Medium	1.23	0.14	0.036	Small
Jump counts of 38+	171.68	< 0.001	0.258	Large	6.35	< 0.001	0.06	Medium	1.26	0.117	0.037	Small
Jump counts of 50+	241.08	< 0.001	0.328	Large	3.08	< 0.001	0.03	Small	2.08	< 0.001	0.06	Medium
CMJ height	173.69	< 0.001	0.327	Large	1.85	0.024	0.025	Small	0.66	0.96	0.027	Small
CMJ depth	113.96	< 0.001	0.241	Large	0.51	0.934	0.007	Small	1.66	0.004	0.065	Medium
Force-based metrics												
Braking RFD	167.39	< 0.001	0.318	Large	6.32	< 0.001	0.081	Medium	0.87	0.705	0.035	Small
Average braking force	257.19	< 0.001	0.418	Large	5.27	< 0.001	0.069	Medium	0.97	0.528	0.039	Small
Peak braking force	206.10	< 0.001	0.365	Large	5.10	< 0.001	0.066	Medium	1.47	0.024	0.058	Medium
Average propulsion force	141.90	< 0.001	0.284	Large	0.70	0.783	0.01	Small	2.07	< 0.001	0.08	Medium
Peak propulsion force	236.02	< 0.001	0.397	Large	3.12	< 0.001	0.042	Small	1.25	0.121	0.05	Small
Velocity-based metrics												
Average braking velocity	11.48	< 0.001	0.031	Small	5.55	< 0.001	0.072	Medium	1.24	0.134	0.049	Small
Peak braking velocity	8.10	< 0.001	0.022	Small	5.09	< 0.001	0.066	Medium	1.39	0.045	0.055	Medium
Average propulsion velocity	49.59	< 0.001	0.122	Medium	4.53	< 0.001	0.06	Medium	0.64	0.966	0.026	Small
Peak propulsion velocity	182.42	< 0.001	0.337	Large	2.10	0.008	0.029	Small	0.71	0.926	0.029	Small

**Table 2:** The daily average total jump counts for athletes across different positions throughout the season, including the number of high-intensity jump counts.

	Middle blockers (MBs)		Outside hitters (OHs)		Opposite hitters (OPPs)		Setters (Ss)	
Total jump counts	84.1 ± 44.9^b,c,d^		59.2 ± 34.2^a,d^		66.5 ± 26.7^a,d^		111.6 ± 61.9^a,b,c^	
Jump counts of 38+	65.4 ± 39.2^b,c,d^	77.8%	39.4 ± 25.9^a,d^	66.6%	47.9 ± 24.1^a,d^	72.0%	19.0 ± 16.6^a,b,c^	17.0%
Jump counts of 50+	39.5 ± 32.7^b,c,d^	44.6%	15.0 ± 15.6^a,c,d^	25.3%	29.7 ± 18.1^a,c,d^	44.7%	0.4 ± 0.8^a,b,c^	0.3%

*Note:* Data are mean ± sd. In addition, the table shows the percentage of high-intensity jump counts relative to total jump counts for each position.

^a^Significantly different from middle blockers; *p* ≤ 0.001 for all.

^b^Significantly different from outside hitters; *p* ≤ 0.001 for all.

^c^Significantly different from opposite hitters; *p* ≤ 0.003 for all.

^d^Significantly different from setters; *p* ≤ 0.001 for all.

**Table 3:** The weekly average CMJ test data for each athlete throughout the season, with force plate metrics averaged weekly for each athlete based on their position providing insights into the positional differences in neuromuscular performance over the course of the season.

	Middle blockers (MBs)	Outside hitters (OHs)	Opposite hitters (OPPs)	Setters (Ss)
CMJ height (cm)	36.1 ± 6.4^b,c,d^	30.0 ± 4.8^a,c,d^	28.0 ± 1.7^a,b^	27.4 ± 2.8^a,b^
CMJ depth (cm)	−41.7 ± 6.4^b,c,d^	−39.6 ± 4.3^a,c,d^	−34.5 ± 3.3^a,b^	−34.5 ± 3.6^a,b^
Braking RFD (N/s)	4617 ± 1959^d^	4449 ± 1228^d^	4193 ± 838^d^	7839 ± 2617^a,b,c^
Average braking force (N)	1335 ± 227^c^	1322 ± 115^c,d^	1181 ± 84^a,b,d^	1698 ± 223^a,b,c^
Peak braking force (N)	1671 ± 322^c^	1648 ± 152^c,d^	1415 ± 96^a,b,d^	2061 ± 248^a,b,c^
Average propulsion force (N)	1351 ± 205^b,c,d^	1286 ± 114^a,c,d^	1102 ± 37^a,b,d^	1446 ± 88^a,b,c^
Peak propulsion force (N)	1712 ± 253^b,c,d^	1649 ± 136^a,c,d^	1399 ± 49^a,b,d^	1994 ± 213^a,b,c^
Average braking velocity (m/s)	−0.98 ± 0.10^c^	−0.96 ± 0.11^c^	−0.91 ± 0.08^a,b,d^	−0.98 ± 0.08^c^
Peak braking velocity (m/s	−1.57 ± 0.20^c^	−1.53 ± 0.20^c^	−1.47 ± 0.14^a,b,d^	−1.60 ± 0.15^c^
Average propulsion velocity (m/s)	1.49 ± 0.08^b,c,d^	1.44 ± 0.13^a,c^	1.34 ± 0.06^a,b,d^	1.44 ± 0.08^a,c^
Peak propulsion velocity (m/s)	2.75 ± 0.22^b,c,d^	2.54 ± 0.17^a,c,d^	2.46 ± 0.07^a,b^	2.43 ± 0.11^a,b^

*Note:* Data are mean ± SD.

^a^Significantly different from middle blockers; *p* ≤ 0.001 for all.

^b^Significantly different from outside hitters; *p* ≤ 0.002 for all.

^c^Significantly different from opposite hitters; *p* ≤ 0.004 for all.

^d^Significantly different from setters; *p* ≤ 0.001 for all.

**Table 4:** Correlations between jumps 50+ and CMJ depth, peak braking force, average propulsion force, and peak braking by position.

Jumps 50+ correlated to	Middle blockers (MBs)		Outside hitters (OHs)		Opposite hitters (OPPs)		Setters (Ss)	
	*r*	*p* value	*r*	*p* value	*r*	*p* value	*r*	*p* value
CMJ depth	−0.444	< 0.001	−0.239	< 0.001	−0.073	0.334	−0.267	< 0.001
Peak braking force	−0.595	< 0.001	−0.01	0.859	−0.033	0.658	0.05	0.452
Average propulsion force	−0.544	< 0.001	−0.249	< 0.001	−0.117	0.119	−0.13	0.051
Peak braking velocity	−0.29	< 0.001	−0.389	< 0.001	−0.091	0.227	−0.217	< 0.001

## Data Availability

The data supporting the findings of this study are not publicly available as they were collected solely for the purposes of this research and contain information that could compromise participant privacy. However, anonymized data can be made available upon reasonable request to the corresponding author, subject to ethical and privacy considerations.

## Supporting Information

Supporting Table 1: Weekly total jump counts, high-intensity jump counts, and force plate metrics throughout the full 16-week season.
