# Supplementary material for: Positional Differences in Jump Loads and Force and Velocity Metrics Throughout a 16-Week Division I Volleyball Season
Source: Transl Sports Med. 2024 Dec 18;2024:5933923. doi: 10.1155/tsm2/5933923 (PMC11669427; doi:10.1155/tsm2/5933923)
Supplement: Supporting Information — Additional supporting information can be found online in the Supporting Information section. [file 5933923.f1.docx]

**Supplemental Table 1.** Jump loads and force plate metrics throughout 16 weeks.

|  | **Week 1** | | | **Week 2** | | | **Week 3** | | | **Week 4** | | | **Week 5** | | | **Week 6** | | | **Week 7** | | | **Week 8** | | |
| --- | --- | --- | --- | --- | --- | --- | --- | --- | --- | --- | --- | --- | --- | --- | --- | --- | --- | --- | --- | --- | --- | --- | --- | --- |
| Total Jump Counts | 77.9 | ± | 36.2 | 69.2 | ± | 36.1 | 74.9 | ± | 53.1 | 67.5 | ± | 39.7 | 83.7 | ± | 43.4 | 63.3 | ± | 40.1 | 80.0 | ± | 45.5 | 89.6 | ± | 48.8 |
| Jump Counts 38+ | 52.0 | ± | 33.1 | 49.1 | ± | 33.6 | 50.8 | ± | 40.3 | 40.2 | ± | 33.0 | 40.0 | ± | 29.2 | 32.8 | ± | 24.8 | 43.4 | ± | 32.3 | 46.7 | ± | 36.4 |
| Jump Counts 50+ | 26.8 | ± | 31.5 | 25.7 | ± | 21.1 | 30.3 | ± | 31.5 | 18.9 | ± | 23.1 | 19.1 | ± | 22.4 | 15.9 | ± | 19.4 | 22.1 | ± | 28.0 | 19.5 | ± | 26.8 |
| CMJ Height (cm) | 30.2 | ± | 6.1 | 30.3 | ± | 5.3 | 33.4 | ± | 7.0 | 30.9 | ± | 5.9 | 32.4 | ± | 6.4 | 30.9 | ± | 5.4 | 31.2 | ± | 5.4 | 30.0 | ± | 5.4 |
| CMJ Depth (cm) | -38.9 | ± | 5.8 | -38.9 | ± | 5.1 | -38.6 | ± | 5.8 | -37.8 | ± | 4.8 | -38.1 | ± | 6.1 | -37.6 | ± | 5.8 | -37.8 | ± | 5.6 | -38.1 | ± | 5.8 |
| Braking RFD (N/s) | 3755 | ± | 1396 | 4204 | ± | 1068 | 4686 | ± | 1523 | 4873 | ± | 1710 | 5750 | ± | 2602 | 5432 | ± | 2489 | 5442 | ± | 2582 | 5549 | ± | 2495 |
| Avg Braking Force (N) | 1248 | ± | 186 | 1301 | ± | 157 | 1325 | ± | 198 | 1358 | ± | 213 | 1424 | ± | 253 | 1411 | ± | 255 | 1402 | ± | 258 | 1426 | ± | 243 |
| Peak Braking Force (N) | 1535 | ± | 245 | 1607 | ± | 210 | 1652 | ± | 263 | 1698 | ± | 265 | 1773 | ± | 317 | 1748 | ± | 311 | 1728 | ± | 319 | 1760 | ± | 302 |
| Avg Propulsion Force (N) | 1268 | ± | 149 | 1275 | ± | 145 | 1320 | ± | 188 | 1327 | ± | 183 | 1354 | ± | 179 | 1341 | ± | 172 | 1332 | ± | 175 | 1331 | ± | 175 |
| Peak Propulsion Force (N) | 1582 | ± | 186 | 1615 | ± | 185 | 1665 | ± | 234 | 1703 | ± | 236 | 1763 | ± | 267 | 1734 | ± | 261 | 1726 | ± | 261 | 1742 | ± | 260 |
| Avg Braking Velocity (m/s) | -0.90 | ± | 0.10 | -0.94 | ± | 0.10 | -0.95 | ± | 0.10 | -0.95 | ± | 0.09 | -0.97 | ± | 0.11 | -0.96 | ± | 0.09 | -0.95 | ± | 0.11 | -0.97 | ± | 0.09 |
| Peak Braking Velocity (m/s | -1.44 | ± | 0.19 | -1.51 | ± | 0.17 | -1.52 | ± | 0.19 | -1.50 | ± | 0.17 | -1.56 | ± | 0.20 | -1.53 | ± | 0.19 | -1.53 | ± | 0.20 | -1.56 | ± | 0.18 |
| Avg Prop Velocity (m/s) | 1.38 | ± | 0.12 | 1.41 | ± | 0.10 | 1.47 | ± | 0.12 | 1.44 | ± | 0.10 | 1.48 | ± | 0.11 | 1.44 | ± | 0.10 | 1.45 | ± | 0.10 | 1.44 | ± | 0.10 |
| Peak Prop Velocity (m/s) | 2.54 | ± | 0.23 | 2.55 | ± | 0.19 | 2.66 | ± | 0.24 | 2.57 | ± | 0.22 | 2.62 | ± | 0.22 | 2.57 | ± | 0.19 | 2.58 | ± | 0.19 | 2.53 | ± | 0.19 |
|  | **Week 9** | | | **Week 10** | | | **Week 11** | | | **Week 12** | | | **Week 13** | | | **Week 14** | | | **Week 15** | | | **Week 16** | | |
| Total Jump Counts | 77.2 | ± | 47.7 | 72.8 | ± | 53.6 | 110.6 | ± | 60.6 | 58.5 | ± | 36.4 | 110.6 | ± | 56.6 | 85.4 | ± | 47.1 | 110.2 | ± | 57.6 | 64.6 | ± | 42.5 |
| Jump Counts 38+ | 43.5 | ± | 35.2 | 40.6 | ± | 37.0 | 58.8 | ± | 43.0 | 29.9 | ± | 25.8 | 58.8 | ± | 32.9 | 43.5 | ± | 20.6 | 62.8 | ± | 36.9 | 36.2 | ± | 27.2 |
| Jump Counts 50+ | 20.0 | ± | 28.9 | 21.3 | ± | 26.3 | 26.4 | ± | 33.3 | 14.0 | ± | 19.1 | 23.6 | ± | 28.5 | 20.0 | ± | 18.8 | 26.7 | ± | 27.0 | 16.9 | ± | 23.5 |
| CMJ Height (cm) | 31.3 | ± | 6.1 | 30.8 | ± | 5.3 | 30.1 | ± | 5.9 | 31.9 | ± | 5.4 | 31.3 | ± | 6.0 | 30.8 | ± | 6.0 | 31.6 | ± | 5.5 | 31.9 | ± | 6.1 |
| CMJ Depth (cm) | -39.4 | ± | 5.6 | -38.4 | ± | 5.3 | -39.9 | ± | 5.3 | -39.6 | ± | 5.8 | -39.6 | ± | 5.8 | -39.6 | ± | 5.3 | -40.4 | ± | 6.4 | -38.9 | ± | 6.1 |
| Braking RFD (N/s) | 5660 | ± | 2198 | 5596 | ± | 2521 | 5117 | ± | 2233 | 6000 | ± | 2460 | 5450 | ± | 2355 | 5208 | ± | 2057 | 5477 | ± | 2429 | 5691 | ± | 2411 |
| Avg Braking Force (N) | 1447 | ± | 232 | 1441 | ± | 260 | 1406 | ± | 246 | 1477 | ± | 260 | 1433 | ± | 266 | 1409 | ± | 240 | 1427 | ± | 259 | 1443 | ± | 258 |
| Peak Braking Force (N) | 1782 | ± | 285 | 1766 | ± | 309 | 1732 | ± | 311 | 1816 | ± | 307 | 1762 | ± | 333 | 1750 | ± | 310 | 1776 | ± | 329 | 1776 | ± | 321 |
| Avg Propulsion Force (N) | 1329 | ± | 159 | 1334 | ± | 172 | 1336 | ± | 177 | 1338 | ± | 170 | 1327 | ± | 175 | 1322 | ± | 165 | 1319 | ± | 174 | 1333 | ± | 168 |
| Peak Propulsion Force (N) | 1765 | ± | 247 | 1757 | ± | 264 | 1741 | ± | 261 | 1785 | ± | 271 | 1751 | ± | 277 | 1748 | ± | 264 | 1764 | ± | 271 | 1763 | ± | 274 |
| Avg Braking Velocity (m/s) | -1.01 | ± | 0.09 | -0.98 | ± | 0.10 | -0.96 | ± | 0.09 | -1.01 | ± | 0.09 | -0.99 | ± | 0.09 | -0.98 | ± | 0.10 | -1.00 | ± | 0.09 | -0.99 | ± | 0.11 |
| Peak Braking Velocity (m/s | -1.62 | ± | 0.16 | -1.59 | ± | 0.18 | -1.55 | ± | 0.18 | -1.64 | ± | 0.17 | -1.60 | ± | 0.18 | -1.57 | ± | 0.18 | -1.61 | ± | 0.17 | -1.59 | ± | 0.20 |
| Avg Prop Velocity (m/s) | 1.47 | ± | 0.10 | 1.45 | ± | 0.10 | 1.42 | ± | 0.11 | 1.49 | ± | 0.11 | 1.45 | ± | 0.11 | 1.44 | ± | 0.11 | 1.46 | ± | 0.10 | 1.47 | ± | 0.11 |
| Peak Prop Velocity (m/s) | 2.58 | ± | 0.22 | 2.56 | ± | 0.19 | 2.54 | ± | 0.21 | 2.60 | ± | 0.19 | 2.58 | ± | 0.21 | 2.56 | ± | 0.22 | 2.59 | ± | 0.20 | 2.60 | ± | 0.22 |

Data are Means ± SD.

The table above shows how jump loads and CMJ metrics recorded on the force plate changed throughout a 16-week season. Significant changes are described for each variable below.

Total Jumps Counts: Week 1 was significantly different than week 12 and 15, *P* ≤ 0.043 for all, and weeks 2, 4, 5, 6 different than week 11, 13, and 15, *P* ≤ 0.001 for all. Week 3 different than week 12 and 13, *P* ≤ 0.29 for all. There were no differences in total jumps between all other weeks, *P* < 0.001 for all.

Jumps Counts 38+: Week 1 was significantly greater than Week 6, 12, and 16 (*P* < 0.001 for all) but significantly less than week 13 (P = 0.022). Week 2 was significantly greater than Week 12 (*P* = 0.005) but significantly less than week 13 (P = 0.029). Week 3 was significantly greater than Week 6, 12, and 16 (*P* ≤ 0.003 for all) but significantly less than week 13 (P = 0.01). Week 5 was significantly less than Week 13 (*P* < 0.001).

Jumps Counts 50+: Weeks 6, 12, and 16 were significantly less than all other weeks during the season, *P* ≤ 0.040 for all. Week 3 produced significantly more 20+ jumps than weeks 4-6, 8-10, 12, and 16, *P* ≤ 0.042 for all. There were no other differences between weeks, *P* ≥ 0.054 for all.

Countermovement Jump (CMJ) Height: Week 3 produced a significantly higher jump height than week 8, *P* < 0.024.

Countermovement Jump (CMJ) Depth: There were no differences between weeks, *P* ≥ 0.313 for all.

Braking Rate of Force Development (RFD): Week 1 was significantly less than weeks 3, 5-10, 12, and 14-16, *P* ≤ 0.029 for all. Week 2 was significantly less than weeks 5, and 12, *P* ≤ 0.034 for all. There were no other differences between weeks, *P* ≥ 0.157 for all.

Average Braking Force: Week 1 was significantly different than all weeks except week 2 and 4, *P* ≤ 0.009 for all. There were no other differences between weeks, *P* ≥ 0.067 for all.

Peak Braking Force: Week 1 was significantly less than weeks 3 and 5-16, *P* ≤ 0.008 for all. There were no other differences between weeks, *P* ≥ 0.134 for all.

Average Propulsion Force: There were no other differences between weeks, *P* ≥ 0.999 for all.

Peak Propulsion Force: Week 1 was significantly less than weeks 5, 9, 12, 15 and 16, *P* ≤ 0.029 for all. There were no other differences between weeks, *P* ≥ 0.066 for all.

Average Braking Velocity: Week 1 was significantly different than weeks 3, 5, 7-10, 12, 13, 15 and 16, *P* ≤ 0.023 for all. There were no other differences between weeks, *P* ≥ 0.084 for all.

Peak Braking Velocity: Week 1 was significantly different than weeks 5, 7-10, 12, 13, 15, and 16, *P* ≤ 0.040 for all. Week 3 and 4 were significantly different than week 12, *P* = 0.046 for all. There were no other differences between weeks, *P* ≥ 0.076 for all.

Average Propulsion Velocity: Week 1 was significantly different than weeks 3, 5, 7, 9, 12, and 16, *P* ≤ 0.016 for all. Week 2 was significantly different than week 12, *P* = 0.009. There were no other differences between weeks, *P* ≥ 0.099 for all.

Peak Propulsion Velocity: Week 3 was significantly different than week 8, *P* ≤ 0.009. There were no other differences between weeks, *P* ≥ 0.157 for all.
